# Arsenite of Copper in Diarrhoea

**Published:** 1891-09

**Authors:** 


ARSENITE OF COPPER IN DIARRHOEA.—Dr. F. Hinz, in the Journal de Med. de Paris, May 3, 1891, strongly advocates the use of arsenite of copper in diarrhoea. He prescribes the drug in the following formula:

R. Arsenite of copper - - g. ss
Water - - - - - - - oz. ij

M. et Sig.: Half a teaspoonful every hour.

During the next day the remedy is only given four times. Recovery is usually rapid.

(We strongly suspect that Dr. Hinz has taken his idea from Dr. John Aulde, of Philadelphia, the latter gentleman having used the drug similarly for some time and having written extensively on the subject.—*The Med. & Surg. Reporter.*)—*St. Louis Cliniqve.*

